# Thermogenic Gene Expression in Human Neck Adipose Tissue in Relation to Circulating and Local Thyroid Hormone Levels

**DOI:** 10.1210/jendso/bvaf178

**Published:** 2025-11-08

**Authors:** Laura Salej Duran, Henrique Camara, Natalia Chaves, Sean D Kodani, Rita Monahan-Earley, Shaelah Huntington, Aaron M Cypess, John M Asara, Yu-Hua Tseng, Benjamin C James, Alina Gavrila

**Affiliations:** Endocrinology Division, Department of Medicine, Beth Israel Deaconess Medical Center, Boston, MA 02215, USA; Department of Integrative Physiology and Metabolism, Joslin Diabetes Center, Boston, MA 02215, USA; Department of Surgery, Beth Israel Deaconess Medical Center, Boston, MA 02215, USA; Department of Integrative Physiology and Metabolism, Joslin Diabetes Center, Boston, MA 02215, USA; Clinical Research Center, Beth Israel Deaconess Medical Center, Boston, MA 02215, USA; Clinical Research Center, Beth Israel Deaconess Medical Center, Boston, MA 02215, USA; Translational Physiology Section, Diabetes, Endocrinology and Obesity Branch, NIDDK, Bethesda, MD 20892, USA; Endocrinology Division, Department of Medicine, Beth Israel Deaconess Medical Center, Boston, MA 02215, USA; Mass Spectrometry Core, Beth Israel Deaconess Medical Center, Boston, MA 02215, USA; Department of Integrative Physiology and Metabolism, Joslin Diabetes Center, Boston, MA 02215, USA; Department of Surgery, Beth Israel Deaconess Medical Center, Boston, MA 02215, USA; Endocrinology Division, Department of Medicine, Beth Israel Deaconess Medical Center, Boston, MA 02215, USA

**Keywords:** TSH, thyroid hormone, thermogenesis, brown adipose tissue, RNA sequencing, mass spectrometry

## Abstract

**Introduction:**

Brown adipose tissue (BAT) contributes to thermogenesis and has been proposed as a therapeutic target for metabolic disease. Thyroid hormones (THs) regulate thermogenic activity, but the relationship between circulating and local TH concentrations and their associations with thermogenic gene and pathway expression in human adipose tissue remain unclear.

**Methods:**

We obtained paired deep neck and subcutaneous adipose tissue samples from adults undergoing thyroid surgery, which represent BAT and white adipose tissue, respectively. Serum and local adipose tissue TH concentrations (T3, T4, TSH) were measured. Bulk RNA-sequencing was performed on adipose tissue samples. Associations between hormone concentrations and thermogenic gene expression and pathway activation were analyzed, with false discovery rate correction for multiple testing.

**Results:**

Both serum and local T4 concentrations were positively associated with thermogenic pathway activation in deep neck adipose tissue. Although serum T3 was also positively associated, local T3 was inversely associated with thermogenic pathways in deep neck adipose tissue. However, circulating TH concentrations did not correlate with local tissue hormone levels. No significant associations were observed between serum or local TH concentrations and individual thermogenic gene expression after correction for different clinical covariates and multiple comparisons.

**Conclusion:**

Local regulation of THs may play a role in human adipose tissue thermogenic activity. Pathway-level transcriptomic analysis may better capture these effects than single-gene approaches. Deep neck adipose tissue can serve as a practical model for studying BAT function and endocrine regulation in humans.

Obesity and diabetes are major global health challenges, driving interest in thermogenic tissues like brown adipose tissue (BAT) as therapeutic targets. Once thought to be limited to rodents and infants, metabolically active BAT has been identified in adults, its activity correlating inversely with visceral adiposity and metabolic disease risk [1-6]. Besides thermogenesis, BAT may support whole body energy expenditure and metabolic homeostasis, making it a promising target for obesity treatment [6].

Thyroid hormones (THs) are key regulators of energy balance and thermogenesis. In rodents, cold exposure robustly activates BAT through sympathetic stimulation and upregulation of local type 2 deiodinase (DIO2), which converts T4 to the active T3, both processes promoting uncoupling protein 1 (UCP1) expression and heat production [7-10]. Similar cold-induced BAT activation occurs in humans [11-13]; however, human BAT is less abundant, more heterogeneous, and embedded heterogeneously within white fat depots [14]. Deep neck fat in adults contains clusters of UCP1-positive brown and beige adipocytes, with thermogenic potential varying by anatomical depth [15-18]. These interspecies differences complicate the translation of rodent findings to human physiology.

Although systemic TH regulates BAT activity in rodents through local actions mediated by transporters, deiodinases, and receptors [7, 19, 20], human responses are inconsistent, and the contribution of local vs circulating TH remains unclear [21-28]. In humans, hyperthyroidism increases skeletal muscle activity, elevating resting energy expenditure and likely reducing the need for BAT-mediated thermogenesis during cold exposure [21-23, 28]. By contrast, in mice, hyperthyroidism markedly enhances BAT thermogenesis, making it a major contributor to excess heat production [29]. In our prior study, BAT activity was observed on positron emission tomography-computed tomography under cold stimulation in both hypothyroid and thyrotoxic states, and it did not consistently increase during the transition from hypothyroidism to thyrotoxicosis [30], suggesting that serum THs may not reflect local TH activity in adult humans.

Here, we evaluated the relationship between circulating and tissue TH concentrations and their associations with thermogenic gene expression in deep (BAT) and subcutaneous (white adipose tissue) neck adipose tissue from adults undergoing thyroid surgery.

## Materials and Methods

**Figure 1.**
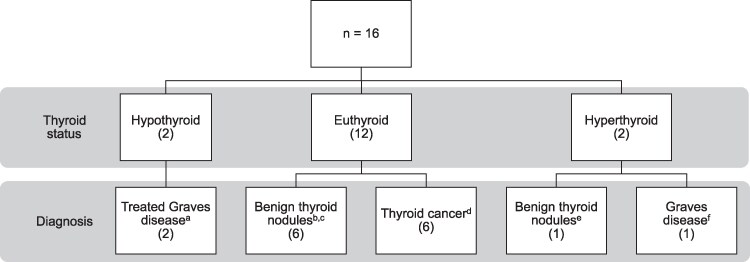
Thyroid status of the study patients at the time of the thyroid surgery. (A) Two patients with Graves’ disease on methimazole who also took Lugol's solution for 10 days before the surgery had thyroid function tests in the overt hypothyroid range at the time of the surgery (TSH 18.17 *µ*IU/L [reference range 0.4-4.5 µIU/L], free T4 0.6 ng/dL [reference range 0.8-1.8 ng/dL], free T3 3.0 pg/mL [reference range 2.3-4.2 pg/mL]; TSH 18.48 µIU/L, free T4 0.4 ng/dL, free T3 2.2 pg/mL), respectively; 1 patient was hypothyroid for 5 weeks, whereas the second patient was euthyroid/hypothyroid for 4 weeks while taking methimazole before starting Lugol's solution before the thyroid surgery. (B) One patient with a hyperfunctioning thyroid nodule/Graves’ disease who was on methimazole and took Lugol's solution for 10 days before the surgery had thyroid function tests within normal range at the time of the surgery (TSH 0.77 µIU/L, free T4 0.8 ng/dL, free T3 2.8 pg/mL); the patient was euthyroid on methimazole for at least 2 months before surgery. (C) One euthyroid patient with benign thyroid nodules had only subcutaneous adipose tissue (no deep adipose tissue) removed during the surgery. (D) One patient who underwent thyroid surgery for thyroid cancer had a minimally elevated TSH of 4.4 µIU/L before the surgery (reference range 0.36-3.7 µIU/L), not on thyroid hormone treatment; because all prior TSH levels were normal, this patient was included in the euthyroid patient group. (E) One patient with an untreated hyperfunctioning thyroid nodule had thyroid function tests in the subclinical hyperthyroid range for at least 1.5 years before surgery; TSH 0.07 µIU/L (reference range 0.4-4.5 µIU/L), free T4 1.2 ng/dL (reference range 0.8-1.8 ng/dL), free T3 3.9 pg/mL (reference range 2.3-4.2 pg/mL) at surgery time. (F) One patient with Graves’ disease took methimazole in the past for only 1 month and then stopped taking this medication 6 months before thyroid surgery; the patient also did not take Lugol's solution, as prescribed before the surgery. Thyroid function tests showed overt hyperthyroidism at the surgery time (TSH 0.02* µ*IU/L [reference range 0.4-4.5 µIU/L], free T4 3.4 ng/dL [reference range 0.93-1.7 ng/dL]).

The protocol was reviewed and approved by the Institutional Review Board at Beth Israel Deaconess Medical Center (BIDMC). We collected deep cervical prevertebral adipose tissue samples from 16 adult patients undergoing thyroid surgery at BIDMC between November 2020 and October 2022. Deep (perithyroidal) and subcutaneous (SC) neck adipose tissues were collected from 15 patients; SC tissue only was collected from 1 patient. Thyroid status and diagnoses of patients can be seen in Fig. 1.

Serum thyroid function tests (TFTs): TSH, free T4 (FT4), and free T3 (FT3) were measured preoperatively by chemiluminescent immunoassays in a fasting state for 9 patients (Quest Diagnostics); when not available, outpatient or immediate postoperative labs were used. Six patients had TFTs measured in an outpatient setting less than 3 months before thyroid surgery for nonfunctional thyroid nodules; they were not taking any thyroid treatment. Serum TSH levels were within normal range for 5 patients, with 1 having a slightly elevated TSH of 4.4 µIU/L (reference range 0.36-3.7 µIU/L). One hyperthyroid patient had TFTs measured during day 1 (<24 hours) after the thyroid surgery; the patient was not on any thyroid treatment before or immediately after the surgery. Serum TSH was available for all, FT4 for 13, and FT3 for 9 participants.

Adipose tissue samples were stored at −80 °C. Tissue T3 and T4 concentrations were measured via targeted liquid chromatography–tandem mass spectrometry using a selected reaction monitoring protocol developed by the BIDMC Mass Spectrometry Core Lab [31, 32]. Quantification was based on external standard curves for T3 and T4, normalized to an internal standard, 13C6-T3[Millipore Sigma] (Supplementary Data Analysis Section) [33]. Adipose tissue TH levels were measured in all 31 samples. All missing values were due to low abundance below the detection limit. Bulk RNA-sequencing (RNA-seq) was performed by Azenta, Inc (Supplementary Data Analysis Section) [33]. A total of 27 neck adipose tissue samples (13 deep, 14 SC) were large enough and well preserved to perform RNA-seq.

## Statistical Analysis

Correlations between serum TSH, FT4, FT3, and tissue T4 and T3 levels were performed using the Spearman rank test, which is relatively insensitive to outliers. We reported the statistical analysis results for the entire patient group. Analysis of the only 9 patients who had serum thyroid tests measured at the surgery time resulted in similar results.

### RNA-seq Data Analysis

Details of the RNA-seq data analysis, including raw reads, gene counts, data normalization, data transformation, gene set enrichment analysis (DESeq2), and reactome pathway analysis (ReactomePA) can be found in the Supplementary Data Analysis Section [33]. Significance was defined at false discovery rate (FDR) < 0.05 using the Benjamini and Hochberg procedure to control the expected proportion of false positives [34]. Normalized enrichment scores (NES) reflect the degree of pathway enrichment adjusted for gene set size and multiple testing.

### Code Availability

The full analysis code is accessible at https://github.com/camara-h/Salej_2025.

## Results

**Figure 2.**
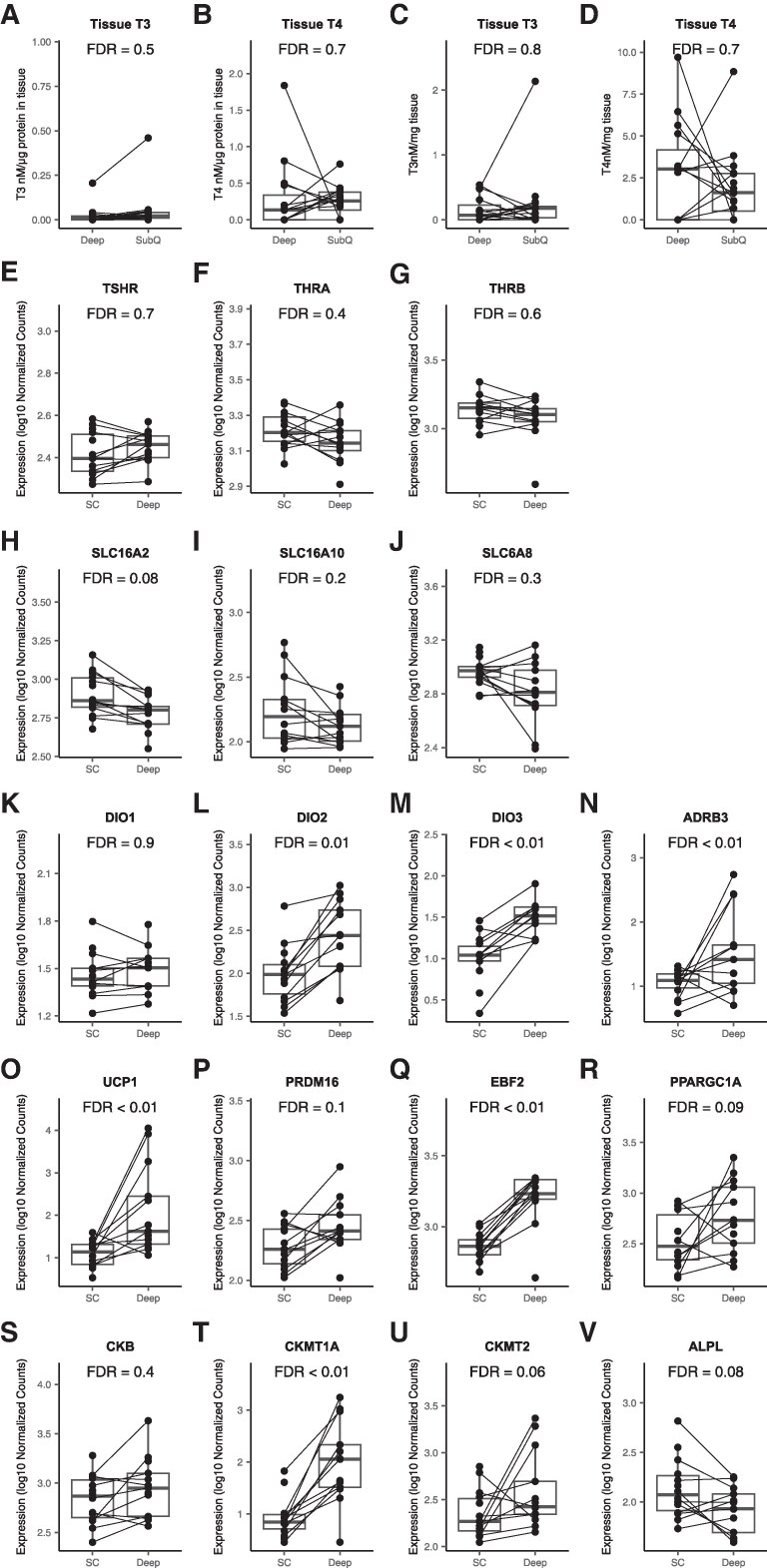
Differences between deep perithyroidal and subcutaneous (SC) neck adipose tissue in: (A) adipose tissue T3 concentration (per microgram of protein in tissue); (B) adipose tissue T4 concentration (per microgram of protein in tissue); (C) adipose tissue T3 concentration (per milligram of tissue); (D) adipose tissue T4 concentration (per milligram of tissue); (E) TSH receptor (TSHR) gene expression; (F) thyroid hormone receptor α (THRA) gene expression; (G) thyroid hormone receptor β (THRB) gene expression; (H) solute carrier family 16 member 2 (SLC16A2) gene expression coding for the thyroid hormone transporter monocarboxylate transporter 8 (MCT8); (I) solute carrier family 16 member 10 (SLC16A10) gene expression coding for the thyroid hormone transporter monocarboxylate transporter 10 (MCT10); (J) solute carrier family 6 member 8 (SLC6A8) gene expression coding for a membrane protein that transports creatine into and out of cells; (K) deiodinase 1 (DIO1) gene expression; (L) deiodinase 2 (DIO2) gene expression; (M) deiodinase 3 (DIO3) gene expression; (N) β-3 adrenergic receptor (ADRB3) gene expression; (O) uncoupling protein 1 (UCP1) gene expression; (P) PR domain-containing 16 (PRDM16) gene expression; (Q) early β-cell factor 2 (EBF2) gene expression; (R) peroxisome proliferator-activated receptor gamma co-activator 1-alpha (PPARGC1A) gene expression; (S) creatine kinase B (CKB) gene expression; (T) creatine kinase mitochondrial 1A (CKMT1A) gene expression; (U) creatine kinase mitochondrial 2 (CKMT2) gene expression; (V) alkaline phosphatase (ALPL) gene expression, encoding tissue nonspecific alkaline phosphatase. Gene expression is reported in transcript per million (TPM). The significance of the difference between deep and subcutaneous neck adipose tissue is expressed as an FDR value above each graph. FDR < 0.05 is considered statistically significant.

Table 1 summarizes the baseline characteristics of the 16 study participants. Among all 16 patients, only 4 were treated for Graves’ disease before the surgery. All 4 patients took Lugol's solution and methimazole for 10 days before surgery, and all had thyroid function tests available at surgery time, which accurately reflected the thyroid status at surgery independently of their prior thyroid condition/treatment. Figure 2 and Table S1 [33] summarize differences in tissue T3 and T4 concentrations and RNA expression of thyroid signaling and thermogenic genes between deep and SC neck adipose tissue. Tissue T4 was measurable in 9 of 15 deep and in 12 of 16 SC adipose tissue samples; 3 patients had both undetectable deep and SC tissue T4 levels. Tissue T3 was measurable in 9 of 15 deep and in 12 of 16 SC adipose tissue samples; only 1 patient had both undetectable deep and SC tissue T3 levels. Tissue T4 concentration was higher than T3 in both deep and SC adipose tissue (T4: 0.29 + 0.12 nM/mg of tissue vs 0.25 + 0.05 nM/mg of tissue, T3: 0.03 + 0.01 nM/mg of tissue vs 0.050 + 0.03 nM/mg of tissue, respectively).

**Table 1.** Baseline characteristics of the study participants (N = 16)

Characteristic	
Age (years)	
Median	52.5
IQR	(34-64)
Sex—no. (%)	
Male	4 (25)
Female	12 (75)
Weight (kg)	
Median	71.8
IQR	(61.1-81.3)
BMI (kg/m^2^)	
Median	25.7
IQR	(22.2-28.3)
Race—no. (%)	
White	12 (75)
Asian	3 (18.8)
African American	1 (6.25)
Coexisting conditions—no. (%)	
Diabetes/prediabetes	2/2 (25)
Hyperlipidemia	8 (50)
Prior medication use—no. (%)	
Levothyroxine	4 (25)
Antidiabetics	2 (12.5)
Statins	1 (6.25)
Fibrates	5 (31.3)
β-blocker	1(6.25)
Serum TSH levels (µIU/L)	
Mean (± SEM)	4.03 (± 1.44)
Range	(0.02-18.48)

Abbreviations: BMI, body mass index; IQR, interquartile range.

### Deep Neck Adipose Tissue Has Higher Thermogenic Gene Expression but Similar Local T3 and T4 Concentrations Compared to SC Neck Adipose Tissue

Although tissue T3 and T4 concentrations did not differ significantly between deep and SC neck adipose depots, thermogenic gene expression was higher in deep tissue, including *UCP1* (FDR < 0.01), *DIO2* (FDR = 0.01), *DIO3* (type 3 deiodinase) (FDR < 0.01), and *ADRB3* (adrenergic beta-3 receptor) (FDR < 0.01). Expression of *CKMT1A* (creatine kinase, mitochondrial 1A), encoding a protein involved in the UCP1-independent thermogenesis (futile creatine cycle) and EBF2 (early B cell factor-2), encoding a transcriptional regulator of brown adipocyte differentiation, were both higher in deep compared to SC adipose tissue (FDR < 0.01) (Fig. 2, Table S1) [33]. A heat map of the top 25 genes and top 10 pathways upregulated in the deep vs SC neck adipose tissue is included as Fig. S1 [33].

### Circulating TH Levels Did Not Reflect Local Neck Adipose Tissue Concentrations

**Figure 3.**
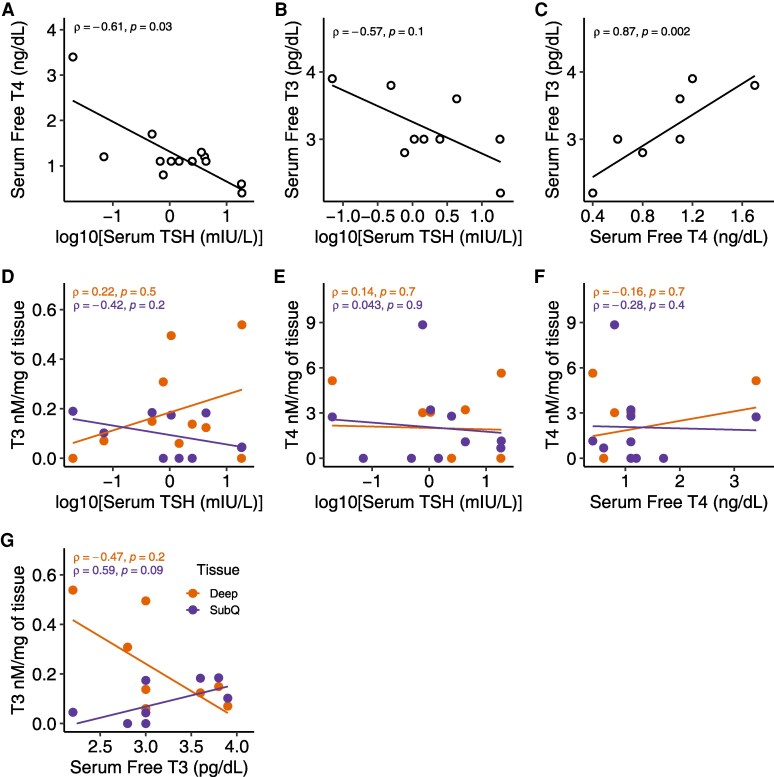
Correlations between serum TSH, T4, and T3, and local adipose tissue T3 and T4 levels. (A) Serum TSH and serum free T4, (B) Serum TSH and serum free T3, (C) Serum free T4 and serum free T3, (D) Serum TSH and tissue T3, (E) Serum TSH and tissue T4, (F) Serum free T4 and tissue T4, (G) Serum free T3 and tissue T3. A *P* < .05 is considered statistically significant.

Although serum TSH and FT4 levels were inversely correlated (ρ = −0.61, *P* = .03), and FT3 positively correlated with FT4 (ρ = 0.87, *P* = .002), circulating TSH, FT3, and FT4 levels did not correlate with their corresponding hormone concentrations in either deep or SC neck adipose tissue (Fig. 3). These findings suggest that systemic TH levels do not reliably reflect local adipose tissue TH status.

**Figure 4.**
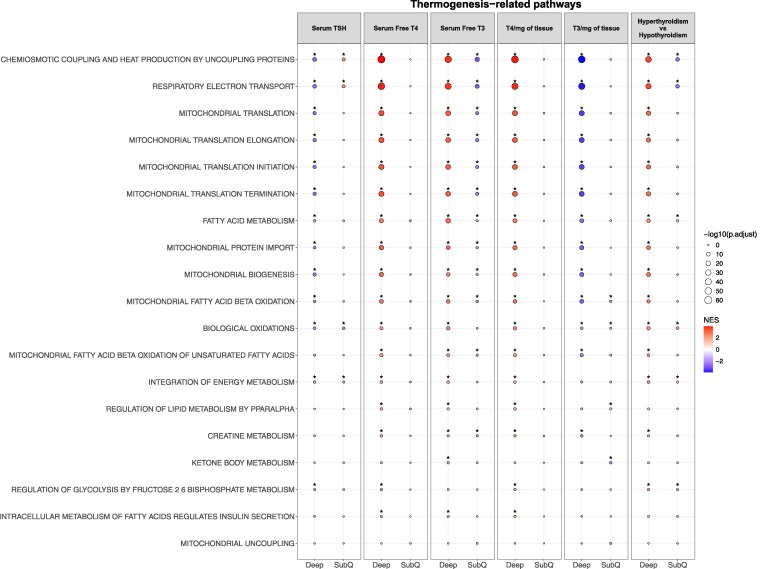
Correlations of thermogenesis-related pathways with serum and local thyroid hormone levels in deep neck and subcutaneous adipose tissue. Fill reflects the sign of the normalized enrichment score (NES). A positive NES indicates pathway upregulation, and a negative NES indicates pathway downregulation.

Thermogenic pathways were upregulated in deep neck adipose tissue and downregulated in SC neck adipose tissue of hyperthyroid as compared to hypothyroid patients, indicating that adipocytes from the deep and superficial neck have distinct responses to the thyroid signaling status (Fig. 4, Fig. S2) [33].

### Serum TSH Levels Are Negatively Associated With Thermogenic Pathways in Deep Neck Adipose Tissue

Serum TSH levels did not correlate with expression of thermogenic genes in either deep or SC neck adipose tissue (Fig. S3) [33]. However, TSH levels were inversely associated with thermogenic pathway activity in deep adipose tissue, including the tricarboxylic acid (TCA) cycle (NES = −2.62, FDR = 1.2 × 10^−5^), thermogenesis by uncoupling proteins (NES = −2.42, FDR = 2.7 × 10^−12^), and respiratory electron transport (NES = −2.59, FDR =8.0 × 10^−11^). Conversely, TSH showed a positive correlation with the TCA cycle pathway in SC adipose tissue (Fig. 4, Fig. S4) [33]. These results suggest that TSH may influence thermogenic activity through modulation of pathways rather than through isolated gene expression changes with opposite effects on thermogenic pathways in deep vs SC adipose tissue.

### Serum Free T4 Levels Positively Correlate With Thermogenic Pathways in Deep Neck Adipose Tissue

**Figure 5.**
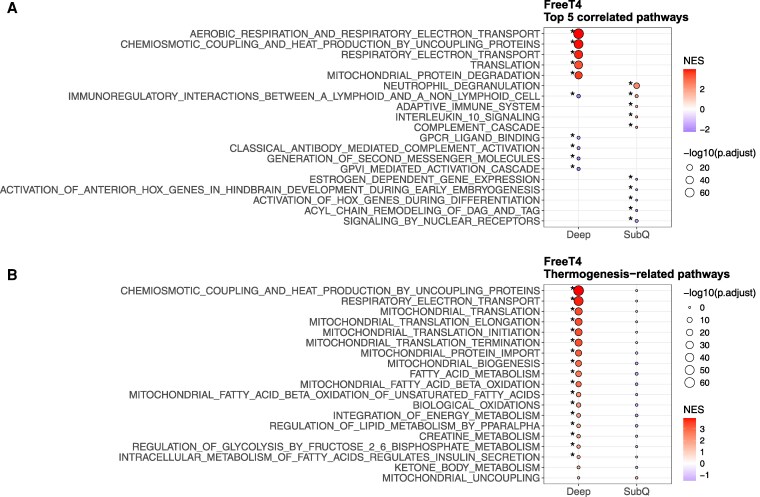
Enriched pathways correlated with serum free T4 concentration. (A) Normalized enrichment score of the top 5 correlated pathways in deep and subcutaneous neck adipose tissue samples. (B) Normalized enrichment score of thermogenic pathways. Fill reflects the sign of the normalized enrichment score (NES). A positive NES indicates pathway upregulation, and a negative NES indicates pathway downregulation. *FDR < 0.05.

We found a significant positive correlation of serum FT4 with thermogenic gene *ADRB3* (*P* = .01, FDR = 0.02, slope = 1.57) and browning gene *PRDM16* (PR domain containing 16; *P* < .001, FDR = 0.047, slope = 0.86) in deep, but not SC neck adipose tissue. There was also a positive correlation with *UCP1* (*P* = .001, FDR = 0.09, slope = 3.29) in deep adipose tissue. No other correlations were noted between serum FT4 and other thermogenic gene expression in either deep or SC adipose tissue (Fig. S3, Table S2) [33]. Pathway analysis revealed positive correlations between serum FT4 and thermogenic pathways in deep neck adipose tissue, including the TCA cycle (NES = 3.03, FDR = 4.6 × 10^−16^), thermogenesis by uncoupling proteins (NES = 3.92, FDR = 3.6 × 10^−61^), respiratory electron transport (NES = 3.81, FDR 1.1 × 10^−50^), and mitochondrial translation (NES = 3.38, FDR = 1.2 × 10^−28^) (Figs. 4, 5), but not in SC adipose tissue. These findings suggest that serum FT4 is associated with enhanced thermogenic activity specifically in deep, but not SC neck adipose tissue.

### Serum Free T3 Levels Positively Correlate With Thermogenic Pathways in Deep Neck Adipose Tissue

**Figure 6.**
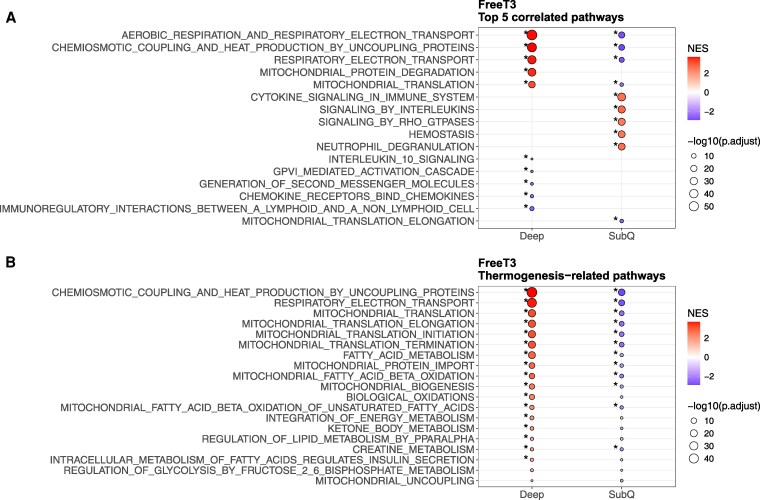
Enriched pathways correlated with serum free T3 concentration. (A) Normalized enrichment score of the top 5 correlated pathways in deep and subcutaneous neck adipose tissue samples. (B) Normalized enrichment score of thermogenic pathways. Fill reflects the sign of the normalized enrichment score (NES). A positive NES indicates pathway upregulation, and a negative NES indicates pathway downregulation. *FDR < 0.05.

Serum FT3 levels did not correlate with thermogenic gene expression in either deep or SC adipose tissue (Fig. S3) [33]. At the pathway level, serum FT3 positively correlated with thermogenic activity in deep neck adipose tissue, including the TCA cycle (NES = 2.7, FDR = 1.5 × 10^−9^), heat production by uncoupling proteins (NES = 3.67, FDR = 1.3 × 10^−48^), respiratory electron transport (NES = 3.60, FDR = 9.5 × 10^−24^), and mitochondrial translation (NES = 3.17, FDR < 0.01). From these pathways, heat production by uncoupling proteins (NES = −1.38, FDR = 0.04) correlated negatively with serum FT3 and SC adipose tissue. (Figures 4, 6). Overall, serum FT3 levels were positively associated with thermogenic pathways in deep neck, whereas a negative association was noted in SC neck adipose tissue.

#### Local T4 positively correlates with thermogenic pathways in deep neck adipose tissue

Local T4 concentrations did not correlate with thermogenic gene expression in either deep or SC adipose tissue (Fig. S3, Table S2) [33]. Thermogenic pathway analysis revealed significant positive correlations with local T4 concentrations in deep adipose tissue, including the TCA cycle (NES = 2.97, FDR = 2.2 × 10^−14^), heat production by uncoupling proteins (NES = 3.89, FDR = 8.6 × 10^−58^), respiratory electron transport (NES = 3.78, FDR = 6.0 × 10^−48^), and mitochondrial translation (NES = 3.38, FDR = 1.2 × 10^−28^) (Fig. 4, Fig. S5) [33]. These associations were not observed in SC adipose tissue. These results suggest that local T4 levels are closely linked to thermogenic pathway activation specifically in deep, but not SC neck adipose tissue.

#### Local T3 negatively correlates with thermogenic pathways in deep neck adipose tissue

Local T3 concentrations did not correlate with thermogenic gene expression in either deep or SC adipose tissue (Fig. S3, Table S2) [33]. Thermogenic pathways were negatively associated with T3 concentrations in deep adipose tissue, including the TCA cycle (NES = −3.01, FDR = 3.9 × 10^−14^), heat production by uncoupling proteins (NES = −3.93, FDR = 3.1 × 10^−58^), respiratory electron transport (NES = −3.84, FDR =2.8 × 10^−49^), and mitochondrial translation (NES = −3.43, FDR = 3.8 × 10^−29^) (Fig. 4, Fig. S6) [33]. These pathways were not associated with local T3 in SC tissue. In summary, local T3 concentrations were inversely associated with thermogenic pathways in deep, but not SC neck adipose tissue.

## Discussion

This is, to our knowledge, the first study to directly quantify T3 and T4 in human adipose tissue while assessing correlations with serum levels and thermogenic gene and pathway expression. Measured tissue TH levels were comparable to those reported in human myocardium [35], supporting assay validity.

First, our findings indicate that circulating TH levels were not correlated with local hormone concentrations in adipose tissue. This suggests that systemic THs may not reliably reflect the hormonal environment within adipocytes. Local regulation through transporter activity, receptor availability, and deiodinase-mediated conversion may influence tissue-specific TH action. Similar patterns have been reported in other tissues, such as the myocardium, where local TH concentrations differ from circulating levels [29].

Second, after correction for different clinical covariates and multiple comparisons, only a few thermogenic genes showed a pattern of positive correlation with circulating and local T4, and a negative correlation with local T3 in deep cervical adipose tissue samples, similar to the pattern seen for thermogenic pathways. However, these correlations were subtle and did not reach statistical significance for most genes. Although THs are known to regulate thermogenic genes such as UCP1 and DIO2 [8, 10, 30], these findings may reflect the complex regulation of thermogenesis, where modest changes across multiple genes are less detectable when assessed individually. These results underscore the limitations of single-gene markers for characterizing BAT activity in human studies, particularly under nonstimulated conditions.

Third, in contrast to the lack of consistent association with single thermogenic gene expression, both serum and local T4 concentrations were positively associated with thermogenic pathway activation in deep neck adipose tissue, whereas local T3 concentrations were inversely associated. One possible explanation for this is the absence of cold exposure in our subjects, which may have limited the conversion of T4 to T3. Although DIO2 expression, which promotes T3 production, was higher in deep adipose tissue, DIO3 expression, which inactivates both T3 and T4 [10, 30, 31], was also elevated. Increased DIO3 activity may have contributed to reduced local T3 availability, favoring T4-associated signaling. The findings suggest that under these conditions, thermogenic activity may be more dependent on T4 and that local tissue regulation of TH metabolism plays an important role.

Significant associations were observed at the pathway level rather than for individual genes suggesting that thermogenic regulation may involve multiple genes and networks. Pathway-level transcriptomic analysis, by aggregating these effects, may provide a more comprehensive and biologically meaningful approach for assessing TH-related thermogenic activity. These results support the consideration of broader transcriptomic strategies in future studies of endocrine regulation of BAT.

Finally, deep and SC neck adipose tissues demonstrated gene expression profiles consistent with BAT and white adipose tissue depots, respectively. These findings suggest that deep neck adipose tissue could serve as a model for studying BAT function, consistent with prior work [15], whereas access to other BAT depots is limited in humans. Together, these depots offer a model for investigating human adipose biology and its hormonal regulation.

This study has limitations, including a modest sample size, clinical heterogeneity, lack of diet/exercise standardization before surgery, and the absence of cold exposure, all of which could influence hormone metabolism and thermogenic activity. Incomplete serum and tissue hormone measurements further limit the analyses, while transcriptomic profiling captures gene expression but not posttranscriptional regulation or protein function. Nevertheless, the application of statistical correction methods and pathway-level analysis strengthens the validity of the associations observed.

In summary, both systemic and local THs, particularly T4, were associated with thermogenic pathway activation in human deep neck adipose tissue, whereas circulating hormone levels did not reflect local tissue concentrations. These findings suggest that local regulation of THs may play an important role in adipose tissue metabolism. Furthermore, pathway-level transcriptomic analysis may better capture the complexity of TH effects than single-gene approaches. Deep neck adipose tissue can serve as a practical model for studying BAT function and endocrine regulation in humans.

## Data Availability

Some or all datasets generated during and/or analyzed during the current study are not publicly available but are available from the corresponding author on reasonable request.

## Abbreviations

BAT: brown adipose tissue
BIDMC: Beth Israel Deaconess Medical Center
DIO2: deiodinase 2
FDR: false discovery rate
FT3: free T3
FT4: free T4
NES: normalized enrichment score
RNA-seq: RNA-sequencing
SC: subcutaneous
TCA: tricarboxylic acid
TFT: thyroid function test
TH: thyroid hormone
UCP1: uncoupled protein 1

## References

[1] Schulz TJ, Tseng YH. Brown adipose tissue: development, metabolism and beyond. Biochem J. 2013;453(2):167‐178. 23805974 10.1042/BJ20130457 PMC3887508

[2] Almind K, Manieri M, Sivitz WI, Cinti S, Kahn CR. Ectopic brown adipose tissue in muscle provides a mechanism for differences in risk of metabolic syndrome in mice. Proc Natl Acad Sci USA. 2007;104(7):2366‐2371. 17283342 10.1073/pnas.0610416104 PMC1892979

[3] Lowell BB, S-Susulic V, Hamann A, et al Development of obesity in transgenic mice after genetic ablation of brown adipose tissue. Nature. 1993;366(6457):740‐742. 8264795 10.1038/366740a0

[4] Singh R, Barrios A, Dirakvand G, Pervin S. Human brown adipose tissue and metabolic health: potential for therapeutic avenues. Cells. 2021;10(11):3030. 34831253 10.3390/cells10113030 PMC8616549

[5] Wibmer AG, Becher T, Eljalby M, et al Brown adipose tissue is associated with healthier body fat distribution and metabolic benefits independent of regional adiposity. Cell Rep Med. 2021;2(7):100332. 34337558 10.1016/j.xcrm.2021.100332 PMC8324464

[6] Cypess AM, Cannon B, Nedergaard J, et al Emerging debates and resolutions in brown adipose tissue research. Cell Metab. 2025;37(1):12‐33. 39644896 10.1016/j.cmet.2024.11.002 PMC11710994

[7] McAninch EA, Bianco AC. Thyroid hormone signaling in energy homeostasis and energy metabolism. Ann N Y Acad Sci. 2014;1311(1):77‐87. 24697152 10.1111/nyas.12374 PMC4451242

[8] Silva JE . Thermogenic mechanisms and their hormonal regulation. Physiol Rev. 2006;86(2):435‐464. 16601266 10.1152/physrev.00009.2005

[9] De Jesus LA, Carvalho SD, Ribeiro MO, et al The type 2 iodothyronine deiodinase is essential for adaptive thermogenesis in brown adipose tissue. J Clin Invest. 2001;108(9):1379‐1385. 11696583 10.1172/JCI13803 PMC209445

[10] Silva JE, Bianco SDC. Thyroid–adrenergic interactions: physiological and clinical implications. Thyroid. 2008;18(2):157‐165. 18279016 10.1089/thy.2007.0252

[11] Van Marken Lichtenbelt WD, Vanhommerig JW, Smulders NM, et al Cold-activated brown adipose tissue in healthy men. N Engl J Med. 2009;360(15):1500‐1508. 19357405 10.1056/NEJMoa0808718

[12] Blondin DP, Labbé SM, Tingelstad HC, et al Increased brown adipose tissue oxidative capacity in cold-acclimated humans. J Clin Endocrinol Metab. 2014;99(3):E438‐E446. 24423363 10.1210/jc.2013-3901 PMC4213359

[13] Chen KY, Brychta RJ, Linderman JD, et al Brown fat activation mediates cold-induced thermogenesis in adult humans in response to a mild decrease in ambient temperature. J Clin Endocrinol Metab. 2013;98(7):E1218‐E1223. 23780370 10.1210/jc.2012-4213 PMC3701264

[14] Liu X, Cervantes C, Liu F. Common and distinct regulation of human and mouse brown and beige adipose tissues: a promising therapeutic target for obesity. Protein Cell. 2017;8(6):446‐454. 28220393 10.1007/s13238-017-0378-6 PMC5445025

[15] Cypess AM, White AP, Vernochet C, et al Anatomical localization, gene expression profiling and functional characterization of adult human neck brown fat. Nat Med. 2013;19(5):635‐639. 23603815 10.1038/nm.3112 PMC3650129

[16] Zingaretti MC, Crosta F, Vitali A, et al The presence of UCP1 demonstrates that metabolically active adipose tissue in the neck of adult humans truly represents brown adipose tissue. FASEB J. 2009;23(9):3113‐3120. 19417078 10.1096/fj.09-133546

[17] Jespersen NZ, Larsen TJ, Peijs L, et al A classical brown adipose tissue mRNA signature partly overlaps with brite in the supraclavicular region of adult humans. Cell Metab. 2013;17(5):798‐805. 23663743 10.1016/j.cmet.2013.04.011

[18] Wu J, Boström P, Sparks LM, et al Beige adipocytes are a distinct type of thermogenic fat cell in mouse and human. Cell. 2012;150(2):366‐376. 22796012 10.1016/j.cell.2012.05.016 PMC3402601

[19] Vella KR, Hollenberg AN. The actions of thyroid hormone signaling in the nucleus. Mol Cell Endocrinol. 2017;458:127‐135. 28286327 10.1016/j.mce.2017.03.001 PMC5592130

[20] Little AG . A review of the peripheral levels of regulation by thyroid hormone. J Comp Physiol B. 2016;186(6):677‐688. 27062031 10.1007/s00360-016-0984-2

[21] Steinhoff KG, Krause K, Linder N, et al Effects of hyperthyroidism on adipose tissue activity and distribution in adults. Thyroid. 2021;31(3):519‐527. 33019884 10.1089/thy.2019.0806

[22] Sun L, Goh HJ, Verma S, et al Metabolic effects of brown fat in transitioning from hyperthyroidism to euthyroidism. Eur J Endocrinol. 2021;185(4):553‐563. 34342595 10.1530/EJE-21-0366 PMC8428075

[23] Zhang Q, Miao Q, Ye H, et al The effects of thyroid hormones on brown adipose tissue in humans: a PET-CT study. Diabetes Metab Res Rev. 2014;30(6):513‐520. 24823620 10.1002/dmrr.2556

[24] Lapa C, Maya Y, Wagner M, et al Activation of brown adipose tissue in hypothyroidism. Ann Med. 2015;47(7):538‐545. 26513396 10.3109/07853890.2015.1085126

[25] Kim MS, Hu HH, Aggabao PC, Geffner ME, Gilsanz V. Presence of brown adipose tissue in an adolescent with severe primary hypothyroidism. J Clin Endocrinol Metab. 2014;99(9):E1686‐E1690. 24915119 10.1210/jc.2014-1343 PMC4154105

[26] Maushart CI, Loeliger R, Gashi G, Christ-Crain M, Betz MJ. Resolution of hypothyroidism restores cold-induced thermogenesis in humans. Thyroid. 2019;29(4):493‐501. 30724123 10.1089/thy.2018.0436 PMC6482913

[27] Broeders EPM, Vijgen GHEJ, Havekes B, et al Thyroid hormone activates brown adipose tissue and increases non-shivering thermogenesis—a cohort study in a group of thyroid carcinoma patients. Ye J, ed. PLoS One. 2016;11(1):e0145049. 26784028 10.1371/journal.pone.0145049 PMC4718641

[28] Maushart CI, Senn JR, Loeliger RC, et al Resting energy expenditure and cold-induced thermogenesis in patients with overt hyperthyroidism. J Clin Endocrinol Metab. 2022;107(2):450‐461. 34570185 10.1210/clinem/dgab706 PMC8764338

[29] Yau WW, Singh BK, Lesmana R, et al Thyroid hormone (T3) stimulates brown adipose tissue activation via mitochondrial biogenesis and MTOR-mediated mitophagy. Autophagy. 2019;15(1):131‐150. 30209975 10.1080/15548627.2018.1511263 PMC6287687

[30] Gavrila A, Hasselgren PO, Glasgow A, et al Variable cold-induced brown adipose tissue response to thyroid hormone status. Thyroid. 2017;27(1):1‐10. 27750020 10.1089/thy.2015.0646 PMC5206686

[31] Yuan M, Breitkopf SB, Yang X, Asara JM. A positive/negative ion-switching, targeted mass spectrometry-based metabolomics platform for bodily fluids, cells, and fresh and fixed tissue. Nat Protoc. 2012;7(5):872‐881. 22498707 10.1038/nprot.2012.024 PMC3685491

[32] Yuan M, Kremer DM, Huang H, et al Ex vivo and in vivo stable isotope labelling of central carbon metabolism and related pathways with analysis by LC-MS/MS. Nat Protoc. 2019;14(2):313‐330. 30683937 10.1038/s41596-018-0102-x PMC7382369

[33] Salej L, Camara H, Gavrila A, Dreyfuss J, Pan H. 2025. Data Repository: Thermogenic Gene Expression in Human Neck Adipose Tissue in Relation to Circulating and Local Thyroid Hormone Levels. Mendeley Data. doi:10.17632/3ycbk7wk8p.3; https://data.mendeley.com/datasets/3ycbk7wk8p/4 PMC12686816 41376652

[34] Benjamini Y, Hochberg Y. Controlling the false discovery rate: a practical and powerful approach to multiple testing. J R Statist Soc. 1995;57(1):289‐300.

[35] Saba A, Donzelli R, Colligiani D, et al Quantification of thyroxine and 3,5,3′-triiodo-thyronine in human and animal hearts by a novel liquid chromatography-tandem mass spectrometry method. Horm Metab Res. 2014;46(9):628‐634. 24591048 10.1055/s-0034-1368717

